# Draft Genome Sequences of Two Novel Sub-Antarctic *Williamsia* Species

**DOI:** 10.1128/genomeA.01047-17

**Published:** 2017-10-12

**Authors:** Akhikun Nahar, Anthony L. Baker, Michael A. Charleston, John P. Bowman, Margaret L. Britz

**Affiliations:** a Tasmanian Institute of Agriculture, University of Tasmania, Hobart, Australia; b School of Physical Sciences, University of Tasmania, Hobart, Australia; c Faculty of Veterinary and Agricultural Sciences, The University of Melbourne, Melbourne, Australia

## Abstract

Illumina MiSeq shotgun sequencing technology was used to sequence the genomes of two novel sub-Antarctic *Williamsia* species, designated strains 1135 and 1138. The estimated genome sizes for strains 1135 and 1138 are 5.99 Mb and 6.08 Mb, respectively. This genome sequence information will aid in understanding the lipid metabolic pathways of cold-tolerant *Williamsia* species.

## GENOME ANNOUNCEMENT

The genus *Williamsia* was described by Kämpfer et al. (1) and belongs to the family *Nocardiaceae* within the order *Corynebacteriales*, phylum *Actinobacteria*. Nine species have been validly published to date (2). To assist in understanding the genetics of cold-tolerant oleaginous *Williamsia* species, the genomes of two *Williamsia* strains, 1135 and 1138, were shotgun sequenced.

The *Williamsia* strains were isolated from soil and detritus from the sub-Antarctic Macquarie Island (54°36′S, 158°54′E) in 2001 and retrieved from the University of Tasmania Antarctic culture collection following lysozyme resistance screening. They were identified by 16S rRNA gene sequencing; BLAST analysis showed 99% similarity to *Williamsia muralis* strain 3541, *Williamsia faeni* strain IHBB 9479, and *Williamsia limnetica* strain L1505. *Williamsia* sp. strains 1135 and 1138 form orange-red colonies on nutrient agar plates, and optimum growth occurs at 20°C and 25°C, respectively. The highest growth rates were observed on fructose, mannitol, and sorbitol for *Williamsia* sp. 1135 and fructose, glycerol, pyruvate, and trehalose for *Williamsia* sp. 1138.

High-molecular-weight genomic DNA was extracted using a modified method of Lévy-Frébault et al. (3) and sequenced using the Illumina MiSeq platform (Macrogen, South Korea). A total of 6,608,962 reads and 1,981,697,652 bp were recorded for strain 1135 and 6,022,802 reads and 1,805,885,539 bp for strain 1138. The whole-genome sequences were assembled using the ABySS *de novo* assembler (4) and annotated using Rapid Annotations using Subsystems Technology (RAST) (5) plus the NCBI Prokaryotic Genome Annotation Pipeline (6).

Strain 1135 possesses a 5.99-Mb genome, with 64.7% GC content, 5,587 coding sequences (CDSs), and 5,388 protein-coding genes; the genome of strain 1138 is 6.08 Mb, with 64.8% GC content, 5,599 CDSs, and 5,513 protein-coding genes. These genomes were similar to those of *W. muralis* (GenBank accession no. NZ_BDAP00000000) and *Williamsia* sp. strain D3 (accession no. NZ_AYTE00000000). However, strains 1135 and 1138 demonstrated 78.78% and 78.91% genetic similarities with *W. muralis* NBRC 105860 (Ga0128692) and 78.68% and 78.54% genetic similarities with *Williamsia* sp. D3 (Ga0041805), respectively, calculated using the average nucleotide identity (ANI) tool of IMG/M (7). A significant separation was also identified for both strains by principal-component analysis (PCA) using IMG/MER with all eight available *Williamsia* genomes; comparatively, the most closely related genomes were those of *W. muralis* and *Williamsia* sp. D3. The two strains are likely distinct novel species, as the genomes are 91.38% similar, below the suggested ANI species demarcation point of above 95% (8) and 96.5% (9).

RAST SEED Viewer 2 identified 442 and 506 genes involved in carbohydrate metabolism and 279 and 361 genes in fatty acid, lipid, and isoprenoid metabolism contained in the strain 1135 and strain 1138 genomes, respectively. Since strain 1138 can utilize glycerol as a sole carbon source and has a diverse lipid biosynthetic capability, it may have potential for biofuel development, which has not been studied previously in any *Williamsia* species.

### Accession number(s).

These whole-genome shotgun projects have been submitted to DDBJ/EMBL/GenBank under the accession numbers MJEI00000000 and MJEJ00000000 for strains 1135 and 1138, respectively.
